# Methodological Challenges and Confounders in Research on the Effects of Ketogenic Diets: A Literature Review of Meta-Analyses

**DOI:** 10.3390/foods13020248

**Published:** 2024-01-12

**Authors:** Katalin Szendi, Edit Murányi, Nicole Hunter, Balázs Németh

**Affiliations:** Department of Public Health Medicine, Medical School, University of Pécs, 7624 Pécs, Hungary

**Keywords:** ketogenic diet, fad diet, types of fatty acids, caloric intake, ketone bodies, literature review, meta-analysis, confounder

## Abstract

Several meta-analyses have found a positive association between a popular type of “fad diet”, ketogenic diets, and their effect on anthropometric and blood parameters. However, the non-specific inclusion criteria for meta-analyses may lead to incorrect conclusions. The aim of this literature review is to highlight the main confounders and methodological pitfalls of meta-analyses on ketogenic diets by inspecting the presence of key inclusion criteria. The PubMed, Embase, and Web of Science databases and the Cochrane Database of Systematic Reviews were searched for meta-analyses. Most meta-analyses did not define the essential parameters of a ketogenic diet (i.e., calories, macronutrient ratio, types of fatty acids, ketone bodies, etc.) as inclusion criteria. Of the 28 included meta-analyses, few addressed collecting real, re-measured nutritional data from the ketogenic diet and control groups in parallel with the pre-designed nutritional data. Most meta-analyses reported positive results in favor of ketogenic diets, which can result in erroneous conclusions considering the numerous methodological pitfalls and confounders. Well-designed clinical trials with comparable results and their meta-analyses are needed. Until then, medical professionals should not recommend ketogenic diets as a form of weight loss when other well-known dietary options have been shown to be healthy and effective.

## 1. Introduction

The classical ketogenic diet was developed in 1921 as an established and effective nonpharmacological treatment option for intractable childhood epilepsy. Since then, several types have been established. A crucial problem is that ketogenic diets, currently classified as “fad diets”, are becoming increasingly popular among those seeking to lose weight [1]. Although, “fad diets” have apparent weight loss effects, there is not enough scientific evidence to determine whether they are healthy and sustainable in the short/long term. Nevertheless, ketogenic diets have been proven to have therapeutic effects on some specific medical conditions, such as refractory epilepsy [2,3,4,5]. In recent decades, research exploring the potential beneficial effects of ketogenic diets for various clinical conditions has become popular, such as neurological disorders (e.g., Alzheimer’s disease, Parkinson’s disease, and amyotrophic lateral sclerosis), type 2 diabetes, obesity, cancer, and more [6,7,8,9,10]. This dietary approach is also quite popular among bodybuilders [11].

Ketogenic diets are characterized by a high fat and very low carbohydrate intake, simulating a state of fasting, which leads to the production of ketone bodies, which cells can use as an energy source. The goal of the diet is to reduce carbohydrate intake to the point where nutritional ketosis is achieved, and then to maintain this state [12]. Nutritional ketosis is a physiological state where ketone bodies in the blood range from 0.5–3.0 mmol/L. Achieving nutritional ketosis typically requires a daily carbohydrate intake of 20–50 grams [13]. In this range, the quantity of ketone bodies is optimal for meeting the energy requirements of the brain and other organs. Furthermore, the blood sugar level remains stable, and minimal insulin is released. However, in diabetic ketoacidosis, the level of ketone bodies in the blood can reach ten-times higher than the previously mentioned range [14,15]. 

Based on macronutrients, the World Health Organization (WHO) considers the following intake proportions as “normal”: carbohydrates should comprise 55–75%, and fats 15–30%, of the total daily energy intake. Saturated fats, they add, should comprise less than 10%, and polyunsaturated fats 6–10%. Monounsaturated fats should constitute the remaining percentage. Finally, proteins should make up 10–15% of the total daily energy intake [16].

Defining the ketogenic diet definitively is not an easy task since several forms of the diet exist. In medical practice and research, the most common variations are the “classic” ketogenic diet [17,18], the modified Atkins [19], and the very low-calorie ketogenic diet [20].

According to the Harvard University definition of a ketogenic diet, 70–80% of the total daily caloric intake should come from fat, 5–10% from carbohydrates, and 10–20% from protein. For a 2000 kcal diet, this means approximately 165 grams of fat, 40 grams of carbohydrates, and 75 grams of protein [21].

The presence of ketosis, achieved through significantly reduced carbohydrate intake (<50 g/day), should be a common characteristic across various ketogenic diets.

The popularity of the diet for both medical and nonmedical reasons can be seen by the significant number of scientific publications. Several meta-analyses have concluded that ketogenic diets might have favorable effects, particularly in type 2 diabetes or overweight patients [22,23,24,25,26]. The results of meta-analyses are at the top of the evidence pyramid and, therefore, they are crucial factors when formulating international recommendations. However, many meta-analyses on the subject contain potentially misleading conclusions. The consequence of these misleading conclusions can be seen in the publication by Evert et al. [27], where the authors suggest that the American Diabetes Association (ADA, 2018) recommends the ketogenic diet as a therapeutic option for type 2 diabetes. In reality, the ADA only mentions the importance of a low-carbohydrate diet (<26%), but there is no mention of a high-fat intake [28].

The hypothesis of this literature review is that randomized controlled clinical trials and, consequently, meta-analyses have several methodological pitfalls, making it even more challenging to establish a clear stance in favor of the diet.

The purpose of this literature review is to draw attention to the methodological challenges commonly encountered in meta-analyses by reviewing the presence of key inclusion criteria and to provide guidance for healthcare professionals to interpret research findings correctly.

## 2. Materials and Methods

The PubMed, Embase, and Web of Science databases and the Cochrane Database of Systematic Reviews were searched for meta-analyses. Search terms included the keyword “ketogenic diet” in the title. The literature search was completed on 13 September 2023. We considered only those meta-analyses that were published in English. For selection, no date restriction was applied.

Inclusion criteria: Only those meta-analyses were included in this literature review that used the term “ketogenic diet” in the title, where the ketogenic diet was not used for epilepsy treatment. Also, the included meta-analyses had to include control diet groups.

Exclusion criterion: Meta-analysis of animal studies.

**Figure 1:**
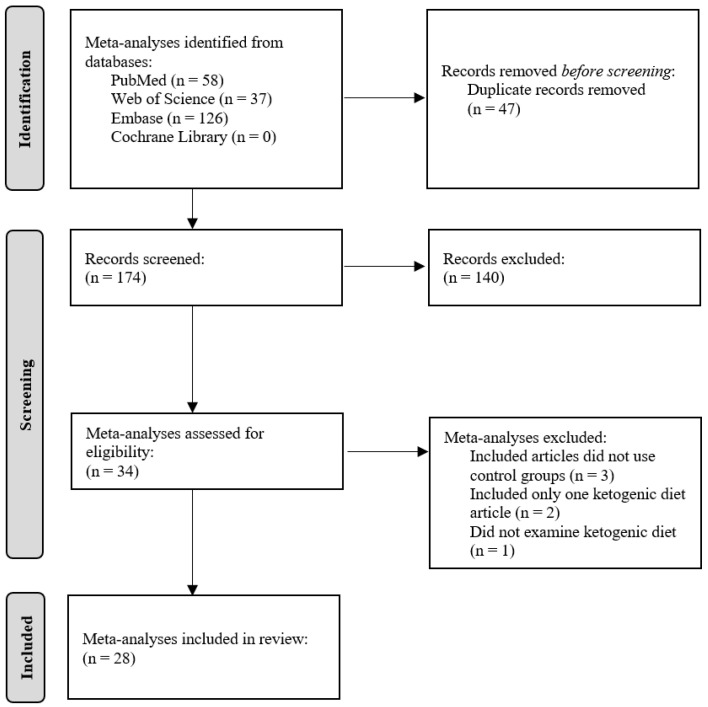
PRISMA flow diagram.

Figure 1 shows the meta-analyses selection process using a PRISMA flow diagram.

Each reference was screened by two independent reviewers based on predefined inclusion criteria. During the full-text analysis, six additional meta-analyses were excluded. Among these, one did not examine the effects of the ketogenic diet, two included only one ketogenic diet article, and three did not have a comparison group with a control group in the included articles. We contacted the authors via the Research Gate website to request the meta-analyses that we did not find. Meta-analyses identified for potential inclusion were reviewed by KSz (Katalin Szendi) and EM (Edit Murányi) independently, with 28 meta-analyses meeting the inclusion criteria and thereby being included in this review. The following data were extracted by KSz and EM and reviewed for extraction accuracy by BN (Balázs Németh): author; year; disease/test parameters; duration (week/month/year); dietary intervention of experimental and control groups; definition of ketogenic diet (g or % carbohydrate); macronutrient ratio (%); calories (kcal); fatty acid type; measurement of ketone bodies; liver and kidney function measurement; results; GRADE (Grading of Recommendations, Assessment, Development, and Evaluations); and risk of bias.

## 3. Results and Discussion

Table 1 shows the presence or lack of inclusion criteria in the included meta-analyses [11,22,23,24,25,26,29,30,31,32,33,34,35,36,37,38,39,40,41,42,43,44,45,46,47,48,49,50] on the effects of ketogenic diets.

The methodological shortcomings of the meta-analyses listed in Table 1, which could have a confounding effect on the results and lead to erroneous conclusions or even the creation of new guidelines regarding the effects of the ketogenic diet, are discussed below. It is important to emphasize that if even one of the included publications in the meta-analysis has methodological flaws, it can influence the final results.

Meta-analyses summarizing the effects of ketogenic diets are considered relatively new. Of the 28 meta-analyses included in this review, 27 were published after 2020, with only one dating back to 2013. However, the majority of the 28 selected meta-analyses did not specify (i) a definition of a ketogenic diet (carbohydrate intake, macronutrient ratios, caloric intake, presence of ketone bodies, etc.), (ii) influencing factors (type of fatty acids), (iii) physiological effects (liver and kidney function), or (iv) comparable factors (defining the parameters of control groups and aligning them with the ketogenic diet group) as inclusion criteria. Twelve out of the 28 did not even specify carbohydrate intake [11,26,29,31,32,33,35,37]. Very few of the meta-analyses collected real dietary data for the ketogenic diet and control groups [22,25,38]. This is significant because, in several studies, the real, re-measured macronutrient ratios shifted away from meeting the criteria of a ketogenic diet. Ketone body levels also support this shift [51,52,53,54,55,56]. Consequently, these meta-analyses interpret their results as those of a non-ketogenic diet rather than the ketogenic diet’s effects.

After reviewing published meta-analyses on the topic, the following discrepancies were identified: 1. Lower caloric intake in the ketogenic diet group than in the control group; 2. The influence of a low (<50 g/day) carbohydrate intake on glucose parameters in the ketogenic diet group; 3. Low carbohydrate intake in the ketogenic diet group but with the inconclusive presence of ketosis; 4. Saturated or unsaturated fatty acid intake in the ketogenic diet group; 5. Whether the prescribed amount of fat/calories was actually consumed or not in the ketogenic diet group; 6. The duration and risks of ketogenic diets.

The Cochrane or other risk-of-bias tools or the GRADE system may face some limitations when assessing these issues. Furthermore, according to Koerich et al. [38], scales have a high degree of subjectivity, so scores can be different when the risk of bias is analyzed by a different research group. This is what could happen in the case of Zhou et al.’s [24] and Rafiullah et al.’s [25] meta-analyses, where more than half of the included articles were the same but conclusions on the quality of evidence were different. Rafiullah et al. collected real, re-measured nutritional data but Zhou et al. did not. Rafiullah et al. seemed to have realized that in their included individual studies, the term “ketogenic diet” referred to low carbohydrate diets, which are different from real ketogenic diets. Therefore, the results of these studies should be examined with caution.

### 3.1. Lower Caloric Intake in the Ketogenic Diet Group than in the Control Group

Naturally, the question arises—how is it possible to compare a low-caloric ketogenic diet with a “normal” or “usual” diet when the measured outcome is weight loss following obesity? One can conclude that a significantly lower caloric intake, which is typical of a ketogenic diet, will, in most cases, lead to weight loss when compared to a “normal” (standard 2000 kcal) caloric intake. Thus, it might falsely appear that the ketogenic diet “outweighs” a “normal” or “usual” diet. The low caloric intake is a confounder. The reality is that, if the caloric intakes are equally low (with a different macronutrient ratio), similar weight loss results can occur. In studies where blood lipid parameters were also examined, along with other cardiovascular risk factors, a new problem arose. Among medical doctors, it is common physiological and pathophysiological knowledge that obesity increases the prevalence of the following cardiovascular risk factors: elevated plasma triglycerides and LDL cholesterol levels; decreased HDL cholesterol levels; increased blood sugar and insulin levels; and elevated blood pressure [57]. If one loses weight, these values tend to improve [58]. A “normal” or “usual” diet control group does not lose weight, so their blood lipid parameters will not change either, which suggests that the low-calorie ketogenic diet is again more effective. This type of study, where the caloric intake is lower in the ketogenic diet group, may mistakenly claim that it is the diet itself and the unrealistic amount of fat and carbohydrate consumption that led to improvements in cholesterol levels. However, we argue that a low caloric intake is more likely to cause weight loss and thus better cholesterol levels.

If the selected meta-analyses are reviewed in terms of caloric intake [11,24,26,29,31,32,34,44,57], several observations can be made. When we further examined the data from the six included publications in Gohari et al.’s meta-analysis [29], we found that information on caloric intake was either completely missing [59] or that the ketogenic and control groups consumed different amounts of calories [60]. The group following the 600–800 kcal ketogenic diet was expected to lose weight more quickly than the low-carb diet group (1400–1800 kcal), meaning their metrics were also expected to be better [60].

In their meta-analysis, Furini et al. [31] pointed out that there is a correlation between obesity and diabetes and lower testosterone levels. So, the question arises—what leads to the improvement in testosterone levels? Is it due to weight loss or the ketogenic diet (as a consequence of low caloric intake)?

Amanollahi et al. [32] included publications in their meta-analysis where there were no prescribed caloric restrictions. This means that caloric intake was not controlled.

Of the five publications selected by Vargas-Molina et al. [11] in their analysis, three had the same planned caloric intake for both the ketogenic and control groups. However, the meta-analysis provided no information on whether the patients actually followed the diet or not.

In the case of Wang et al.’s meta-analysis [34], two of the included publications [61,62] prescribed more calories for the ketogenic group than for the control group.

In their meta-analysis, Zhou et al. [24] suggested that low-calorie ketogenic diets had a favorable effect on overweight patients’ weight control. However, this is true for almost any diet with a low-calorie content. According to the results of Zhou et al. [24], the ketogenic diet (and not the low-caloric ketogenic diet) had a favorable effect on weight control. This can be a misleading conclusion even for non-specialized readers.

The definition of the ketogenic diet in Yang et al.’s analysis [44] did not include caloric intake. In the analysis by Lee et al. [45], one of the selected studies [63] had a significant difference in caloric intake between the two diet groups (ketogenic diet: 1035 ± 808 kcal, control group: 3157 ± 808 kcal).

Lee et al. [26] argued that in patients with obesity, the main obstacle to participating in weight-loss programs was usually caloric restriction; however, the studies they included had no caloric restrictions for the ketogenic diet. This could seem attractive to those who are concerned about caloric restrictions. Does this mean that overweight participants who previously consumed 3000–5000 kilocalories a day can switch to a ketogenic diet and still consume the same amount? In one of the included publications [64], the ketogenic diet group was provided with pre-prepared meals, while the control group prepared their meals based on prior recommendations. This implies that the pre-prepared main and supplementary meals had caloric restrictions if the group with the pre-prepared meals could not eat anything else in the meantime.

In their meta-analysis, Muscogiuri et al. [46] set a very low caloric intake (≤800 kcal/day) as an inclusion criterion for the ketogenic diet. However, there was hardly any information provided about the control diets, which was present in only eight out of the 15 included publications. With such low caloric intake, it cannot be definitively stated that the ketogenic diet was better than any other diet in terms of weight loss if there was no proper control group consuming the same low caloric intake. However, the authors concluded that the ketogenic diet was recommended for overweight individuals.

Castellana et al. [49] compared very low-caloric ketogenic diet groups with higher caloric intake control groups in their meta-analysis. Their final conclusions were mostly based on before-and-after analysis, mainly due to the absence of control groups.

The analysis by Bueno et al. [50] was one of the very first meta-analyses on the ketogenic diet that this review selected, dating back to 2013. Incomplete inclusion criteria were also a pitfall of the 2013 article, although information on caloric intake was essential for weight loss endpoint assessment, especially in both groups. As a result, long-term weight loss was observed in the ketogenic diet, overweight group.

### 3.2. Influence of Low (<50 g/day) Carbohydrate Intake on Glucose Parameters in the Ketogenic Diet Group

Studies that examined type 2 diabetes and the ketogenic diet in comparison to a “normal”/“usual” diet control group when assessing blood sugar levels and the improvement of insulin resistance are also problematic. If the ketogenic diet allows for only 50 g/day of carbohydrates in patients with diabetes compared to the usual 325 g/day carbohydrate intake for patients with diabetes [65], which group is expected to experience a decrease in blood sugar levels and the reversal of insulin resistance—those groups consuming fewer carbohydrates or those consuming as much as before? Once again, the ketogenic diet appeared to be superior, erroneously. In such cases, it is difficult to create a control group where both groups consume similarly low levels of carbohydrates. In these situations, it is worth comparing the ketogenic diet with known, proven beneficial diets, such as the Mediterranean diet, the DASH diet (Dietary Approach to Stop Hypertension), a vegetarian diet, a vegan diet, or a lower carbohydrate diet (<325 g/day but >50 g/day). Furthermore, in a ketogenic diet, the consequence of low carbohydrate intake is low fiber intake, which may increase the risk of colorectal cancer [66,67,68,69,70,71].

### 3.3. Low Carbohydrate Intake in the Ketogenic Diet Group but with the Inconclusive Presence of Ketosis

In this case, a ketogenic diet, where the real carbohydrate intake was higher than 50 g/day, was compared to a control group, and ketone levels in the blood were not monitored. If ketone levels were monitored, patients were not identified to be in nutritional ketosis [51]. However, these studies were labeled as ketogenic diets in the meta-analysis [22]. If there is no sustained, constant ketosis, the diet cannot be considered a ketogenic diet, otherwise erroneous conclusions can be made. Rafiullah et al. [25] came to the same conclusion.

Regarding carbohydrate intake and the presence of nutritional ketosis in the included meta-analyses [22,23,24,25,31,32,33,34,36], the following issues arose.

Furini et al. [31] stated in the introduction that the carbohydrate intake in a ketogenic diet was typically <30 g (<6%); however, they included an article [72] where the carbohydrate intake in a normocaloric ketogenic diet was 15%, which does not meet the criteria of a ketogenic diet.

Of the 18 publications included by Amanollahi et al. [32], one [73] had too much carbohydrate (25%) in the ketogenic diet. In this case, the results obtained likely do not reflect the effects of the ketogenic diet. While Amanollahi et al. [32] did not explicitly define the ketogenic diet, they mentioned that in 5 out of the included 18 studies, ketone levels in the blood were measured. The blood ketone levels increased by an average of 0.56 mmol/L compared to the start of the ketogenic diet (and to reiterate, the range for nutritional ketosis is 0.5–3.0 mmol/L). This suggests that in these five studies, some participants might not have been in a state of ketosis.

In the meta-analysis by Parry-Strong et al. [22], who found a low risk of bias (Cochrane Risk of Bias Tool version 2), five out of eight included publications showed that the ketogenic diet groups actually consumed more carbohydrates than are necessary to induce ketosis [51,52,53,54,55]. Therefore, the results from the two-year interventions, which included three studies [51,52,74], might not be reliable. Another confounder was the glucose-lowering medications taken by the participants, which can influence the HbA1c levels in interventions lasting longer than six months for patients with prediabetes and type 2 diabetes.

Zhao et al. [33] collected data on ketone bodies from their included articles. As a result, they found that the levels of ketone bodies were not significantly higher in the ketogenic diet groups compared to the control diets. This comparison implies that neither or both groups were in ketosis.

In the meta-analysis by Wang et al. [34], the carbohydrate intake values from the individual studies might not have necessarily induced nutritional ketosis; however, the meta-analysis did not specifically address this.

In some of the included articles [53,75] in Li et al.’s [23] analysis, the diets utilized did not meet the criteria of a ketogenic diet. Although the authors regarded the included studies as “high quality” (Jadad scale), it should be noted that, based on the real carbohydrate intakes, participants were most likely not in a state of nutritional ketosis.

The meta-analysis by Zhou et al. [24] did not consider the real carbohydrate intake data available from the individual articles [55,56]. This is probably why the authors rated the articles as “high quality” (Cochrane Handbook).

In the analysis by Rafiullah et al. [25], the aforementioned problems were repeated [52,53,55,56]; the ketogenic diet groups actually consumed more carbohydrates than necessary to induce ketosis. Therefore, the authors did not think it appropriate to interpret the results as an effect of the ketogenic diet.

In the meta-analysis by Ashtray-Larky et al. [36], previously mentioned issues were accompanied by the fact that in one of the included publications [76], the group following the ketogenic diet consumed 40% protein, which could be too high to maintain ketosis. Excess protein intake can lead to the production of glucose through gluconeogenesis, reducing the production of ketone bodies [77].

### 3.4. Saturated or Unsaturated Fatty Acid Intake in the Ketogenic Diet Group

The unfavorable cardio-metabolic effects of saturated fatty acids have been known for a while in contrast to certain types of unsaturated fatty acids [78]. There are types of the ketogenic diet that prioritize the consumption of unsaturated fatty acids [79] but most do not pay attention to this. Rather, the ketogenic diet recommends saturated animal-origin fats. If a meta-analysis does not take this into account, it may include both types, or it may not even be clear in the individual studies which type of fat the ketogenic diet group consumed. Thus, new erroneous results on lipid parameters may emerge because of this confounder. Greene et al. [80] even assured concerned participants in the ketogenic diet group that such a high consumption of saturated fat is not harmful.

Regarding fat consumption, the included meta-analyses [22,29,35,36,44] reveal the following.

Gohari et al. [29], as well as Ashtray-Larky et al. [36], included two studies [60,75] that, in total, provided 10 g of olive oil to the ketogenic diet group and defined it as “daily fat intake”. This does not meet the criteria of a ketogenic diet as this constitutes approximately 90 kcal; when compared to the daily recommended 600 kcal, this only represents 15% of fat intake. It was not typical of the studies included in the meta-analyses to recommend unsaturated fatty acids since mainly saturated fatty acids appear in ketogenic diets. Therefore, in the case of these two articles, the consumption of olive oil should be evaluated as a positive aspect.

Since the included meta-analyses in this review did not deal with the type of fatty acids, the final results might be combined and distorted.

In the analysis by Parry-Strong et al. [22], three of the included studies [52,74,81] had more than 50% of the patients receiving lipid-lowering drugs. This can lead to distortion of the results in the case of measured TG and HDL levels.

In the case of Amini et al.’s [35] meta-analysis, the only thing defined as a criterion for including the articles listed in Table 1 was the amount of fat intake in the ketogenic and control diets. This seems insufficient for the ketogenic diet (>45%), especially when compared to the average energy intake. These data were not provided for the 23 articles that Amini et al. included in the meta-analysis [35].

Yang et al. [44] chose a definition for ketogenic diets that did not specify the amount of fat intake.

### 3.5. Whether the Prescribed Amount of Fat/Calories Was Actually Consumed or Not in the Ketogenic Diet Group

In randomized controlled clinical trials, which generally form the basis of meta-analyses, patients’ energy, fat, and carbohydrate consumption are not always closely monitored. Therefore, it is possible that subjects in a ketogenic diet group following a normocaloric diet may not be able to consume as much fat and, during the study, are in a state of caloric restriction. In studies that monitored prescribed vs. real caloric intake and macronutrient ratios in the ketogenic and control groups, it was observed that there were significant differences in many cases. As a result, participants in the ketogenic diet group are likely to fall out of the range of nutritional ketosis [51,52,53,54,55]. Only two meta-analyses collected real nutritional data from individual studies [25,36].

### 3.6. Duration and Risks of Ketogenic Diets

The long-term effects of a ketogenic diet can be unfavorable; however, there are very few meta-analyses including studies that track patients following a ketogenic diet for multiple years. Participants often find it difficult to adhere to the prescribed diets for even up to two years [51,52,74]. The risk of the long-term consumption of high amounts (>7%) of saturated fatty acids can lead to the development of cancer and cardiovascular diseases. The lack of vegetables, fruits, and grains can eventually result in fundamental micronutrient deficiencies and an increased risk of cancer [82]. Prolonged excessive fat metabolism can damage liver function over the long term or exacerbate existing problems. Excessive protein consumption can burden the kidneys. Low fiber intake can lead to constipation. Maintaining a state of nutritional ketosis with low carbohydrate intake can lead to confusion and irritability [83]. However, if clinical trials were planned to span several years, they could reveal the potential long-term adverse effects of ketogenic diets and monitor liver and kidney function.

Among the included meta-analyses, only one collected study lasted longer than 6 months [22]. However, in such long-term interventional trials, patients were unable to maintain the prescribed diet and, therefore, the results did not describe the effects of ketogenic diets. Several meta-analyses examined ketone bodies [22,26,29,32,33,44] or described the results of liver and kidney function [32,33,49] tests, but those were not treated as inclusion criteria.

### 3.7. Ketogenic Diet and Cancer

An interesting area for studies related to ketogenic diets is their effect on patients with malignant tumors. According to preclinical research results, this area holds promise. The most intriguing question for patients with malignant tumors following a ketogenic diet would be whether or not any changes occurred in the cancerous tissue and any indicators. The meta-analyses included in this literature review did not seek answers to this question [32,33,35,44]; the study endpoints were mostly weight loss and blood sugar or lipid parameters. In one case, tumor markers were also examined [44], but, overall, there was no difference in the two groups following different diets.

In the meta-analyses conducted by Amanollahi et al. [32], patients with malignant tumors lost weight, and their glucose, IGF-1, and triglyceride levels decreased by the end of the ketogenic diet. However, insulin, CRP, cholesterol, liver, and kidney function values did not change. It is not clear from the results whether these changes were relative to patients’ previous states or whether they were within normal limits. Additionally, these values were not compared to a control group. Ultimately, Amanollahi et al. [32] believed that weight loss did not pose a problem for non-cachexic cancer patients.

Based on the meta-analysis by Taftian et al. [35], patients with malignant tumors were able to lose weight due to the ketogenic diet. However, unfortunately, weight loss is a common side effect of malignant tumors.

### 3.8. Limitations

A systematic review of randomized controlled trials (RCTs) would provide a more detailed, comprehensive picture of the effects of ketogenic diets. With this approach, it would become clear whether any studies have actually paid attention to important aspects (such as the aforementioned pitfalls present in many of the studies) during the investigation of the ketogenic diet. Gathering these RCTs could potentially lead to a more credible meta-analysis with more reliable results. Another limitation of this literature review is that only those meta-analyses were included in which the title mentioned the diet of the experimental group as the “ketogenic diet”; it is important to acknowledge that other terms or phrases referring to the ketogenic diet might exist. Nonetheless, the primary goal of this literature review was to provide a brief, thought-provoking overview of the methodological challenges in the included 28 meta-analyses and to identify gaps in the existing meta-analysis literature.

## 4. Conclusions

In everyday medical practice, one of the cornerstones of decision-making is the knowledge of reliable, systematically processed scientific evidence. To achieve this, reliable results reported in systematic reviews and meta-analyses are essential. However, when it comes to the study of ketogenic diets, the extremely heterogeneous data present challenges. Despite various methodological shortcomings, over two-thirds of the aforementioned meta-analyses reported positive results in favor of ketogenic diets, which can be misleading for both professionals and the general public seeking to lose weight. Taking into account the inclusion criteria mentioned above, well-designed clinical studies with comparable results, and systematic analyses that summarize them, are needed. Until then, for the purpose of controlling one’s weight, ketogenic diet types are not recommended when there are many other proven effective and healthy dietary choices available.

## Figures and Tables

**Table 1:** The presence or lack of inclusion criteria in the meta-analyses.

Reference	Disease/Test Parameters	Duration (Week/Month/Year)	Dietary Intervention (Experimental–Control Group)	Definition of KD (g/% Carbohydrate)	Macronutrient Ratio (%)	Calories (kcal)	Fatty Acid Type	Measurement of Ketone Bodies	Liver and Kidney Function Measurement	Outcome
Gohari et al. [29]	Serum uric acid	>2 weeks	KD–NIC	NIC	NIC	NIC	NIC	Achieving a state of ketosis, but no further data	NIC	0
Du et al. [30]	Components of metabolic syndrome	3–52 weeks	KD (all doses and all forms)–comparable diet	NIC	NIC	NIC	NIC	NIC	NIC	+
Furini et al. [31]	Testosterone level	4–14 weeks	KD–any	Any KD protocol	Any KD protocol	Any KD protocol	Any KD protocol	NIC	NIC	+
Amanollahi et al. [32]	Cancer (body composition, glucose and lipid profile)	10 days–6 months	KD–standard diet	NIC	NIC	NIC	NIC	NIC, but examined	NIC, but examined	+
Parry-Strong et al. [22]	NIDDM, pre-diabetes (anthropometry, glucose and lipid profile)	>6 months	VLCh-KD–control diet (>50 g/nap/>10% carbohydrate)	≤50 g/day/ ≤10%	NIC	NIC	NIC	NIC, but data collection	NIC	+(/0)
Zhao et al. [33]	Cancer (body composition, glucose and lipid profile)	1–20 weeks	KD–variable	NIC	NIC	NIC	NIC	NIC, but examined	NIC, but examined	+(KD is safe, weight loss occurred)
Vargas-Molina et al. [11]	Athletes (muscle mass)	>8 weeks	KD–ND	NIC	NIC	NIC	NIC	NIC	NIC	+/−
Wang et al. [34]	Athletes (body composition)	>2 weeks	LCh-KD–non-LCh-KD	<50 g/day/<5%	NIC	NIC	NIC	NIC	NIC	0
Li et al. [23]	NIDDM (glucose and lipid profile)	>2 months	VLCh-KD–diabetes diet	<50 g/day/<10%	NIC	NIC	NIC	NIC	NIC	+
Zhou et al. [24]	NIDDM, overweight (weight loss, glucose and lipid profile)	3 months–2 years	KD–other types of diet	<50 g/day	NIC	NIC	NIC	NIC	NIC	+
Rafiullah et al. [25]	NIDDM (body weight, glucose and lipid profile)	>12 weeks	VLCh-KD–recommended diet	<50 g/day/<10%	NIC	NIC	NIC	NIC	NIC	+(But not recommended)
Taftian et al. [35]	Cancer (weight loss)	1–24 weeks	KD–any	NIC	NIC	NIC	NIC	NIC	NIC	+(Weight loss occurred)
Ashtary-Larky et al. [36]	Athletes/prescribed training (body composition, anthropometry)	>2 weeks	KD–non-KD	<50 g/day	NIC	NIC	NIC	NIC	NIC	+
Amini et al. [37]	Body composition, anthropometry	2–96 weeks	KD–control diet	NIC	KD: fat > 45%, control: fat < 30%	NIC	NIC	NIC	NIC	+
Koerich et al. [38]	Athletes/trained adults (performance, body composition)	4 days–12 weeks	KD–non-KD	<50 g/day/<10%	KD: carbohydrate < 10%, fat > 60–80%; control: carbohydrate ≥ 40%	NIC	NIC	NIC	NIC	0
Luo et al. [39]	NIDDM (body weight, glucose and lipid profile)	0.57–48 weeks	KD–non-KD	NIC	NIC	NIC	NIC	NIC	NIC	+
Zaki et al. [40]	NIDDM (body weight, glucose profile)	ND	KD–control diet	NIC	NIC	NIC	NIC	NIC	NIC	+/−
Zaki et al. [41]	NIDDM (body weight, glucose profile)	ND	KD–control diet	<10–50 g/day	NIC	NIC	NIC	NIC	NIC	+/−
López-Espinoza et al. [42]	Obesity (anthropometry, glucose and lipid profile)	>2 weeks	KD–VLED/LFD	<10%	NIC	NIC	NIC	NIC	NIC	0
Cao et al. [43]	Endurance Athletes (aerobic capacity and exercise performance)	5 days–12 weeks	LCh-KD–non-LCh-KD	<10%	NIC, but detailed data collection	NIC, but data collection	NIC	NIC	NIC	0
Yang et al. [44]	Cancer (body weight, glucose and lipid profile, tumor markers, side effects)	4–24 weeks	LCh-KD–any non-KD	<50 g/day/<10%	High fat, moderate protein, very low carbohydrate	NIC	NIC	NIC, but examined	NIC	0
Lee et al. [45]	Athletes (body fat percentage, respiratory exchange rate, total cholesterol)	1–24 weeks	KD–NIC	<50 g/day	NIC, but detailed data collection	NIC, but data collection	NIC	Yes	NIC	0
Lee et al. [26]	Overweight, obesity + training program (waist circumference, TG)	4 weeks–6 months	LCh-KD–ND	NIC	NIC	No caloric restriction	NIC	NIC, but examined	NIC	+
Muscogiuri et al. [46]	Obesity (body composition, glucose and lipid profile)	3 weeks–24 months	VLE-KD–NIC	30–50 g/day	KD: Carbohydrate 13–25%, protein 40–45% (0.8–1.2 g/bwkg/day), fat 40–45%	≤800 kcal/day	NIC	NIC	NIC	+
Acharya et al. [47]	Kidney stones	ND	KD–NIC	NIC	NIC	NIC	NIC	NIC	NIC	−
Choi et al. [48]	Overweight or obesity ± NIDMM	120 min–1 year	KD–several LFD	5–10%	NIC	NIC	NIC	NIC	NIC	+
Castellana et al. [49]	Overweight or obesity (anthropometry, blood pressure, glucose and lipid profile, liver, and kidney function)	3 weeks–2 years	VLE-KD–non-VLE-KD	<30–50 g/day	NIC, but data collection	NIC, but data collection	NIC, but data collection	NIC	NIC, but data collection	+
Bueno et al. [50]	Obesity (long-term weight loss, blood pressure, glucose and lipid profile)	12–24 months	VLCh-KD–LFD	<50 g/day/<10%	control: fat < 30%	NIC, but data collection	NIC	NIC	NIC	+

Abbreviations: KD, ketogenic diet; LCh-KD, low carbohydrate ketogenic diet; LFD, low fat diet; ND, no data; NIC, not an inclusion criterion; NIDDM, non-insulin-dependent diabetes mellitus; TG, triglyceride; VLCh-KD, very low carbohydrate ketogenic diet; VLED, very low energy diet; VLE-KD, very low energy ketogenic diet. Outcomes are categorized as favorable (+), unfavorable (−), or neutral (0) with respect to the ketogenic diet.

## Data Availability

Data is contained within the article.
